# Phytochemical Fingerprinting of Nigerian Versus South African *Loranthus micranthus* Linn. Extracts and Their Antimicrobial Potentials Against Five Resistant Bacteria

**DOI:** 10.3390/molecules31193489

**Published:** 2026-10-01

**Authors:** Siyabonga Hlophe, Kokoette Bassey

**Affiliations:** Department of Pharmaceutical Sciences, School of Pharmacy, Sefako Makgatho Health Sciences University, Pretoria 0208, South Africa; siyabonga.hlophe@smu.ac.za

**Keywords:** phytochemical fingerprinting, antibacterial activity, mistletoe tree, thin-layer chromatography (TLC), ultra-high-performance liquid chromatography–mass spectrometry (UPLC-MS), minimum inhibitory concentrations (MIC)

## Abstract

**Background:** *Loranthus micranthus* has been traditionally used in ethnomedicine for managing diabetes, hypertension, and schizophrenia. In response to the escalating issue of antibiotic resistance and the desire for alternatives with fewer side effects, attention has shifted towards natural medicines. This study investigated phytochemical fingerprinting and antibacterial activity of the plant from both Nigeria and South Africa because samples from both countries are classified as African. **Methods**: Standard techniques, including thin-layer chromatography and ultra-high-performance liquid chromatography–mass spectrometry, were used for chemical fingerprint profiling. Minimum inhibitory concentrations assay evaluated the antimicrobial potential of extracts and standards. **Results**: Notably, thin-layer chromatography analysis unravelled an identical chemical signature for samples from both countries. Distinct colours indicated the presence of anthracene derivatives, coumarins, and phenolic carboxylic acids, among others. The ultra-performance liquid chromatography–mass spectrometry results mirrored that of the thin-layer chromatography, that is, identical chemical signature and identified peaks including but not limited to stearic acid, quercetin, isoreserpin, and tiamulin in the dichloromethane extracts. Meanwhile, the methanol extracts exhibited compounds like 4-hydroxyhygric acid, quercetin, and catechin-3’-glycoside, among others. The antibacterial activity against *Pseudomonas aeruginosa*, *Staphylococcus aureus*, *Bacillus strains*, *Escherichia coli*, and *Streptococcus pyogenes* afforded minimum inhibitory concentration values of 1.5 × 10^−2^ mg/mL for all the bacterial strains except *Streptococcus pyogenes* with minimum inhibitory concentration = 7.5 × 10^−3^ mg/mL when compared with ciprofloxacin positive control with a minimum inhibitory concentration = 0.0039 mg/mL. **Conclusion:** The apparent mono-directional thin-layer chromatography, ultra-performance liquid chromatography–mass spectrometry chemical signature, as well as the minimum inhibitory concentration values obtained from all the samples supported the classification of *Loranthus micranthus* Linn from both countries as African, and the extracts have the potential to inhibit resistant *Pseudomonas aeruginosa*, *Staphylococcus aureus*, *Bacillus strains*, *Escherichia coli*, and *Streptococcus pyogenes* species.

## 1. Introduction

According to the World Health Organization (WHO) approximately 17% of confirmed bacterial infections globally were resistant to antibiotic interventions in 2023, thus highlighting the incessant global burden of antimicrobial resistance (AMR). As recorded, antimicrobial resistance increased by greater than 40% of the pathogen–antibiotic combinations monitored by WHO between 2018 and 2023, with an average annual increase of 5–15% [1].

With regards to the impact of AMR on human mortality rate, WHO estimated 4.95 million deaths in 2019 and more than 4.7 million deaths globally in 2021, including approximately 1.27 million deaths directly attributable to bacterial AMR. Consequentially increasing treatment costs, intensive-care requirements, additional diagnostic procedures and the use of more expensive second-line antibiotics have an enormous negative impact on the socioeconomic well-being of the global populace. WHO estimated that, under current trajectories, treatment of resistant bacterial infections could cost approximately US$412 billion annually by 2035, with an additional US$443 billion per year in productivity losses. These trends underscored the indispensable urgent need for the discovery of new antibacterial agents and structurally diverse compounds from natural sources, including medicinal plants such as *Loranthus micranthus* Linn (*L. micranthus*) [2].

The identification of disease-causing microorganisms, necessitating unique treatment and preventive techniques, highlights the need for increased pharmaceutical research and development from various sources. Indigenous plants, having long been utilised for medicinal purposes in ancient African civilisations and beyond [3] may hold promise in this regard. Compounds derived from mistletoe have demonstrated noteworthy antimicrobial and medicinal significance due to a rich profile of bioactive secondary metabolites that are able to interfere with several pathogenic pathways. Examples of mistletoe bioactive phytosecondary metabolites include flavonoids, viscotoxins, lectin, and terpenoids [4]. The antimicrobial significance of mistletoe can never be overemphasised. Studies have consistently demonstrated that ethanolic and methanolic mistletoe extracts displayed high potency against Gram-positive bacteria such as *Staphylococcus aureus* and *Bacillus subtilis*, while generally less sensitive towards Gram-negative strains like *Escherichia coli* and yeasts like *Candida albicans* [5].

The detection of disease often relies on identifying defective molecules or abnormal ratios of normal molecules in bodily fluids that may be indicative of faulty metabolism. Investigating these molecules and their interactions in both healthy and unhealthy conditions assist in diagnosis and therapy monitoring. To conduct such studies, it is necessary to first identify, measure, and purify the molecules in question.

Phytochemical analyses of plants with a rich historical background have frequently resulted in the isolation of bioactive compounds [4]. Therefore, it is crucial to establish a fingerprint for each extract in a larger collection to assess its stability over time. Preferably, this technique should be based on electronic data storage, retrieval, and analysis [5].

Throughout recorded history, humanity has faced exposure to a wide variety of microorganisms’ antimicrobial resistance and virulence [6]. Quorum sensing inhibitor is an effective strategy to attenuate the virulence and drug resistance of *Pseudomonas aeruginosa*, among other pathogens. Consequently, there is an imperative need for novel antimicrobial agents to address these challenges [7]. In the ongoing search for solutions, plants such as *L. micranthus* have emerged as one of the most common sources of antimicrobial agents, playing a crucial role in providing potential remedies to combat microbial threats.

*L. micranthus* (*L. micranthus*) is an African mistletoe belonging to the *Loranthaceae* family and native to eastern Nigeria and other regions of Africa. Mistletoes are semi-parasitic plants that grow on various host trees and shrubs that provide them with minerals, food and water while producing their own carbohydrates via photosynthesis [8]. *L. micranthus* is found in Nigeria and South Africa, with various local names depending on the region, primarily thriving in the southeastern part of Nigeria on host trees such as *Persia americana*, *Baphia nitida*, *Kola acuminata*, *Pentaclethra macrophylla*, and *Azadirachta indica* [8].

The plant has been used in traditional medicine in Nigeria and other African regions to treat conditions such as diabetes, hypertension, and schizophrenia and as an immune booster [9,10]. *L. micranthus* extracts were found to contain a variety of phytoconstituents and compounds. A crude methanolic extract of *L. micranthus* leaves from *P. americana* was found to include terpenoids, steroids, oils, proteins, resins, flavonoids, tannins, saponins, alkaloids, reducing sugar, acidic compounds, glycosides, and carbohydrates, with alkaloids being the most abundant.

The chemical composition of *L. micranthus* has been observed to be seasonal. Methanolic extracts of *P. americana* leaves harvested in April had more carbohydrates, acidic compounds, steroids, and alkaloids than those gathered in July when the wet season was at its optimum. Tannins, saponins, glycosides, and reducing sugars were not found in samples taken in July. However, higher quantities of flavonoids and oils were found in July samples compared to the April ones.

Previous research has shown that the composition and biological activities of mistletoe are affected by the harvesting season and host tree species, dictating its phytochemical components and bioactivity [10,11]. Besides the influence of the host tree on the phytochemical composition and potency of the biological activities of the comparative mistletoe tree, other factors such as harvesting season, soil type (geographically) for cultivation, and harvesting age were reported to influence the measured activities [11,12].

We previously reported on the comparative study of Nigerian and South African mistletoe, but the safety, standardised dosing, and comprehensive in vivo mechanisms and antimicrobial comparison evaluations are still unknown for *L. micranthus* (African mistletoe) from both South Africa and Nigeria [13].

In this study, we investigated and compared their phytochemical fingerprinting and evaluation of antimicrobial potentials against selected *Pseudomonas aeruginosa*, *Staphylococcus aureus*, *Bacillus strains*, *Escherichia coli*, and *Streptococcus pyogenes* resistant [14] species.

## 2. Results

### 2.1. Percentage Yield of NGDCM, SADCM, NGMeOH, and SAMeOH Extracts

For the extraction yield, the NGDCM and NGMeOH sample afforded 1.80% and 4.31%, respectively. The SADCM and SAMeOH sample on the other hand resulted in a percentage yield of 5.08% and 7.15%, respectively.

### 2.2. Phytochemical Fingerprinting of NGDCM vs. SADCM by Thin-Layer Chromatography

**Figure 1:**
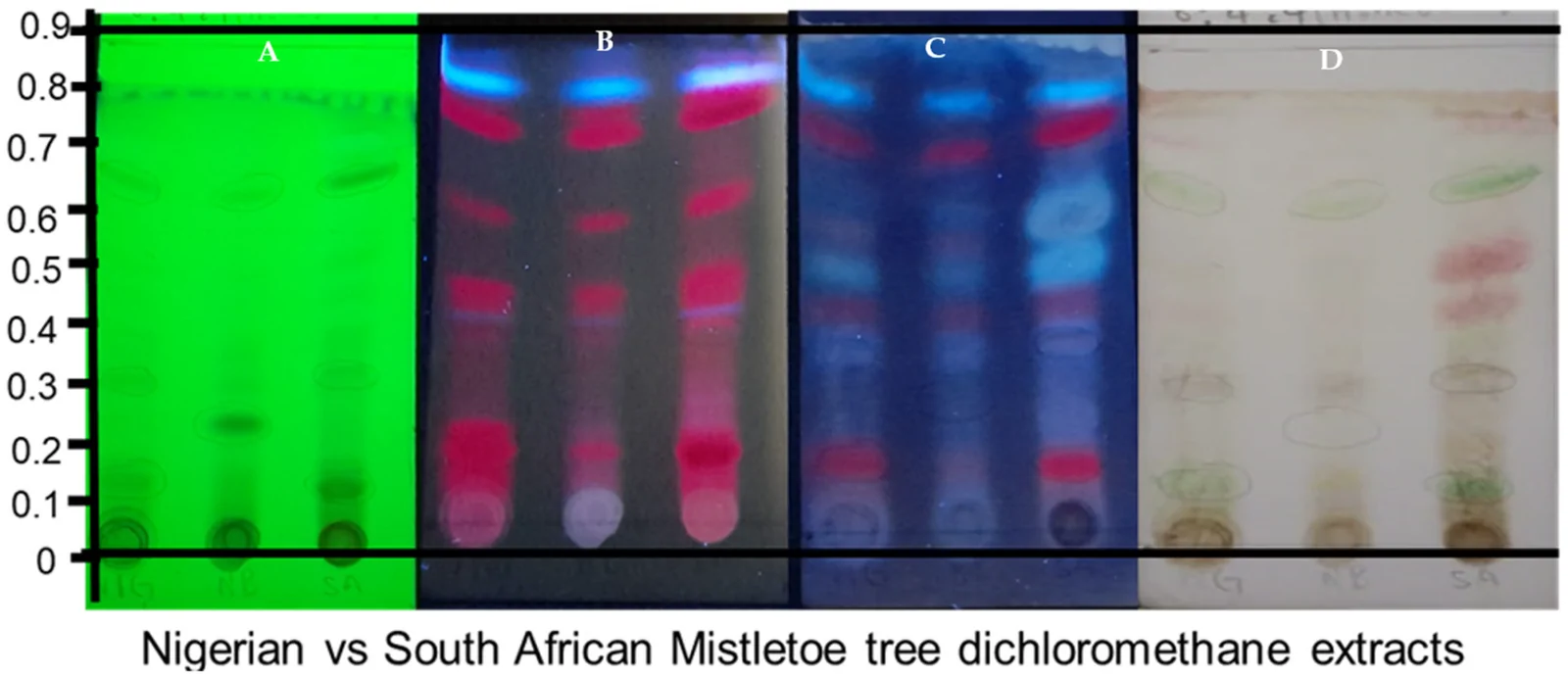
TLC chromatogram revealing the fingerprints of the DCM extracts. Track 1 = Nigerian Green (NGG); 2 = Nigerian Brown (NGB), and 3 = South African (SA) mistletoe extracts. TLC mobile-phase n-haxane: acetone: ethyl acetate (8:4:4 *v*/*v*/*v*) viewed at 254 nm (**A**), 366 nm (**B**), derivatised with PEG solution (**C**) and derivatised with 9: 1 MeOH: H_2_SO_4_ *v*/*v* (**D**).

The Nigerian samples consisted of Nigerian green (NGG) and Nigerian brown (NGB). NGG was obtained from a greenhouse, while NGB was obtained from a sun-dried plant sample. The reason for having these two samples was to investigate the effect of different drying methods on the phytochemical constituents of the extracts. Unfortunately, this parameter could not be investigated for the South African sample due to the COVID-19 national lockdown restrictions, which negatively affected sample collection. TLC plates were injected with standard solutions and chromatographed with various mobile phases. The results demonstrated that the mobile-phase hexane: acetone: ethyl acetate (8:4:4) (*v*/*v*/*v*) provided a strong resolution for the DCM extracts. The fingerprinting/banding patterns of Nigerian brown and Nigerian green were identical to those of the South African extract, as shown in Figure 1.

TLC plates often contain a fluorescent indicator that causes the plate to glow green when exposed to UV light at a wavelength of 254 nm (less frequently at 365 nm with a mercury lamp). Substances that absorb UV light in the corresponding wavelength area will quench the green fluorescence, resulting in dark purple or bluish patches on the plate [15].

Chemical reactions can be performed on the TLC plate, as shown in the following steps. More accurate findings can be achieved by spraying or dipping certain reagents (or by subsequent heating), which produce coloured or fluorescent compounds with the substances to be evaluated [15]. The colour shift visible during TLC visualisation is caused by an interaction between the spraying reagent and the extract solution spotted on the plate. The intensity of colour is usually affected by the concentration of the extract on the plate. As a result, colour change is solely used to identify the distinct constituents based on their reactivity with the spraying reagent. If the spray reagent is modified, the same extract may produce a different and distinct colour. If you examine the plate in UV before spraying, you may see a completely different picture of the plate since only UV-sensitive components will be visible [16].

Polyphenols are a major class of higher plant secondary metabolites. It comprises several well-known subgroups such as flavonoids, phenolic acids, of natural colours. Polyphenols found in fruits, vegetables, spices, wines, and drinks (tea, coffee, and juice) are an essential element of the daily human diet due to their vast variety of biological activities. They are naturally occurring antioxidants with redox characteristics that operate as radical scavengers, reducing agents, and hydrogen donors [17].

The developed plates were dried at room temperature and then sprayed with polyethylene glycol (PEG) reagent to visualise for the presence or otherwise of the different phytochemical groups. For compounds that could not be visualised via derivatisation, the UV lamp was used to make them visible. Coumarins and phenolic carboxylic acids were detected from distinct fluorescence quenching of all coumarins and phenolic carboxylic acids at UV 254 nm, while viewing at 365 nm afforded intense blue or green fluorescence for simple coumarins and phenolic carboxylic acids. However, yellow, brown, blue, or blue-green fluorescence were indicators of furano and pyrano coumarins. Natural products-polyethylene glycol (NP/PEG) reagent intensified and stabilised the existing fluorescence of the coumarins. Phenolic carboxylic acids fluoresce as blue or blue green coloration.

As evident in Figure 1 plate A and viewed at 254 nm, distinct fluorescence quenching of all coumarins, phenolics, alkaloids, flavonoids, and anthracene derivatives was observed. But, from Figure 1 plate B, which was viewed under 365 nm, distinct red, fluorescent colour bands were observed at Rf values of 0.15, 0.45, 0.6, and 7.5. According to [17], this could signify the presence of all anthracene derivatives at varying degrees, depending on the colour intensity. On the other hand, at Rf value 0.41 and 0.84, a blue colour fluorescent indicated the presence of alkaloids, flavonoids, coumarins, and phenolic carboxylic acids as possibly present in the SADCM and NGDCM mistletoe extracts.

As for Figure 1, plate C, sprayed with PEG and dried for 5 min after being visualised at 365 nm, two colours dominantly appeared under UV with different intensities which were blue at Rf 0.25, 0.36, 0.5, 0.6, 0.7, and 0.84 and red at Rf 0.2, 0.45, and 0.75. The red colours signified the presence of anthracene derivatives, and the blue colours proved the presence of coumarins and phenolic carboxylic acids [17]. The result in Figure 1, plate D (fourth) that was derivatised with MeOH: H_2_SO_4_ (9:1 *v*/*v*) showed distinct colours at different Rf values. At Rf value 0.13, the band appeared green for SA and NG extracts, implying the presence of coumarins and phenolic carboxylic acids, and the red colour at Rf 0.6 and 0.7 indicated the presence of saponins [18]. These bands were only present in the SADCM extracts and not the NGDCM extracts, thus highlighting the marginal variations among the samples.

### 2.3. Phytochemical Fingerprinting of NGMeOH vs. SAMeOH by Thin-Layer Chromatography

**Figure 2:**
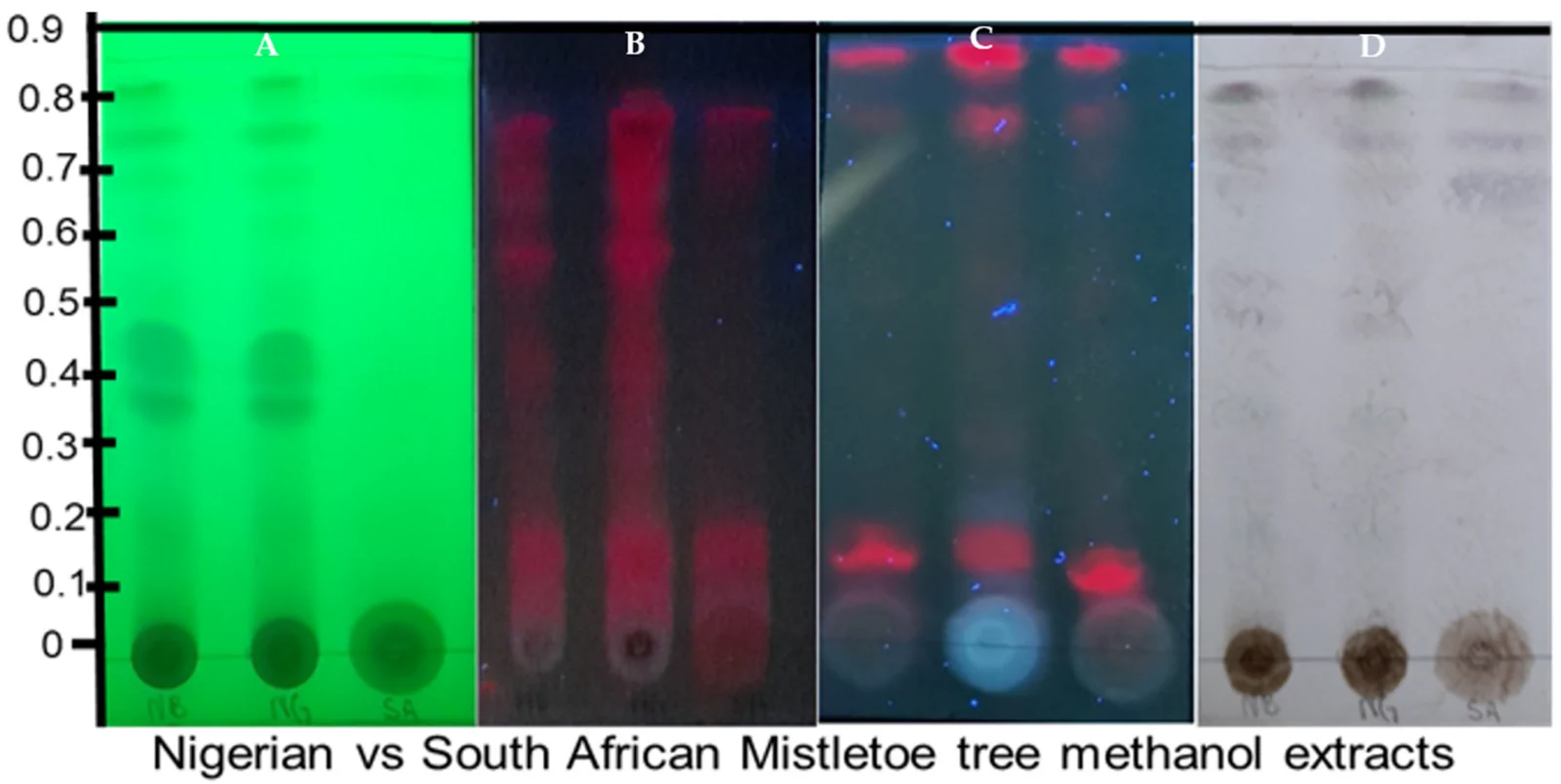
TLC chromatogram revealing the fingerprints of Nigerian and South African mistletoe tree MeOH extracts. TLC mobile phase: EtOAc: toluene (1:2 *v*/*v*) viewed at 254 nm plate (**A**), 365 nm (**B**), derivatised with PEG (**C**) and derivatised with methanol: H_2_SO_4_ (9:1 *v*/*v*) (**D**).

Just as was the case with dichloromethane extract, the TLC phytochemical fingerprinting was conducted for the methanol extracts for samples from Nigeria and South Africa to generate a chemical signature for the polar extracts. The fingerprint afforded distinct compound bands at Rf values of 0.34, 4.5, 0.6, 0.68, 0.75 and 0.82 (Figure 2). A thorough look at Figure 2, plate A, observed under 254 nm, revealed the distinct fluorescence quenching of all coumarins, phenolic, alkaloids, flavonoids, and anthracene derivatives because these compounds do not absorb energy at this wavelength to display colours [17]. But, when the same plate was captured at 365 nm (plate B), all the compound bands absorbed UV energy and subsequently fluorescence with red colour compound bands, thus indicating the presence of all anthracene derivatives in the NGMeOH extracts at Rf = 0.17, 0.6, 0.75, and 0.82 and in the SAMeOH extract at only Rf = 0.17 and 0.82. One could also observe traces of a light blue compound band in Nigerian samples to agree with a similar marginal variation in the fingerprint of the DCM extracts.

The phytocompounds with blue colour in the Nigerian sample were better visualised when the plate was derivatised with PEG and then allowed to dry. The compound bands appeared at Rf of 0.05 and 0.2; Figure 2, plate C. This colour is typical of coumarins and phenolic carboxylic acids. On the other hand, both the NGMeOH and SAMeOH at the Rf value of 0.17, 0.75 and 0.82. showed a red colour for presence of anthracene derivatives [17] even with this derivatising agent. However, when the TLC plate was derivatised with H_2_SO_4_:MeOH (1:9 *v*/*v*), purple/violet colour compound bands appeared at Rf of 0.6, 0.75, and 0.82 to suggest the presence of saponins [18]. The observation that different classes of phytochemical compound band appeared at different UV wavelengths or when different derivatising agents were applied on the developed TLC plates supported the fact that diverse visualisation methods played a vital role with regards to compound identification via TLC analysis.

### 2.4. Phytochemical Fingerprinting of NGDCM vs. SADCM by UPLC-MS

**Figure 3:**
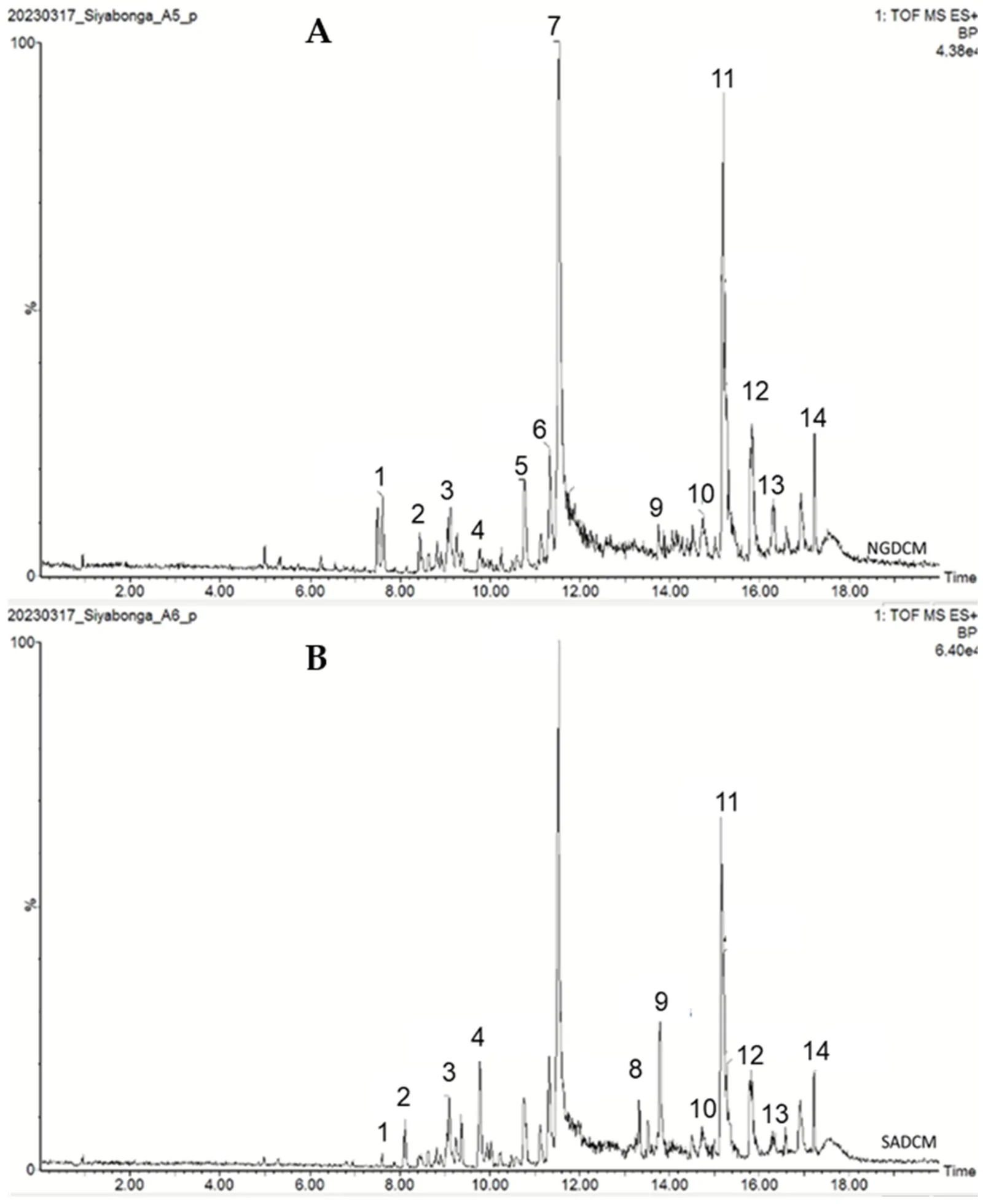
The total ion chromatograms of NGDCM (**A**) and SADCM (**B**) under the positive ionization mode.

The phytochemical fingerprinting of *L. micranthus* Linn. from Nigeria and South Africa from the TLC perspective suggests an identical signature with very minute and almost negligible differences. The fact that TLC, though affordable among its other advantages, is characterised by very low detection and sometimes gives a false positive warrant that results obtained from it be confirmed with a high-resolution instrument [19]. Factoring in on the latter, the same extract solutions used for the TLC studies were analysed with high-resolution UPLC-MS and the result is depicted in Figure 3.

The UPLC-MS chromatogram revealed a total of 14 peaks for NGDCM and 14 peaks for SADCM (Figure 3). While both extracts share common peaks, a distinct but minor peak at 13.32 min (Peak 8) in SADCM is in support of the very meagre difference highlighted from the TLC study. Peaks at 14.74 min (Peak 10) and 16.25 min (Peak 13) exhibited the lowest intensity in both extracts. Interestingly, the peak at 6.60 min (Peak 1) shows moderate intensity in NGDCM but low intensity in SADCM, and the peak at 9.79 min (Peak 4) shows moderate intensity in SADCM and the lowest intensity in NGDCM. The highest intensity peak at 11.53 min (Peak 7) and 15.19 min (Peak 11) was consistent in both extracts, indicating a shared major or marker compound.

These peak intensities correlate with compound concentrations, thus implying that there are two main marker compounds in samples of *L. micranthus* from Nigeria and South Africa at an Rt of 11.53 min and 15.25 min. The absence of the 13.32 min peak in NGDCM may suggested a lower to zero concentration of the corresponding compound compared to SADCM; discrepancies in peak intensity and the presence or absence of peaks in crude extracts are not always apparent and necessitate the application of multivariate data analysis approaches for thorough scrutiny [20]. The major compounds at 11.53 and 15.19 min show similar high abundance in both extracts. The unique compound in SADCM at 13.32 min could be a pharmacological or toxicological indicator from samples from both countries. Hence, its identity is crucial.

### 2.5. Identification of Marker Compound Peaks in NGDCM vs. SADCM

In the analysis of UPLC-MS chromatogram for NGDCM and SADCM extracts, the identification of peaks was conducted using a combination of Mass Bank and literature references. However, it is essential to note that the identification process faced limitations due to the relatively scarce research on NG and SA *Loranthus micranthus*, particularly concerning the dichloromethane (DCM) extract. Peaks were initially matched against the Mass Bank database, a comprehensive repository of mass spectra and associated chemical information. This database allowed for the identification of compounds based on their mass-to-charge ratio (m/z) and spectral characteristics. In cases where Mass Bank did not provide conclusive identifications or lacked data for specific compounds, literature references were consulted. Published studies on *L. micranthus* were reviewed to aid in the identification process.

**Figure 4:**
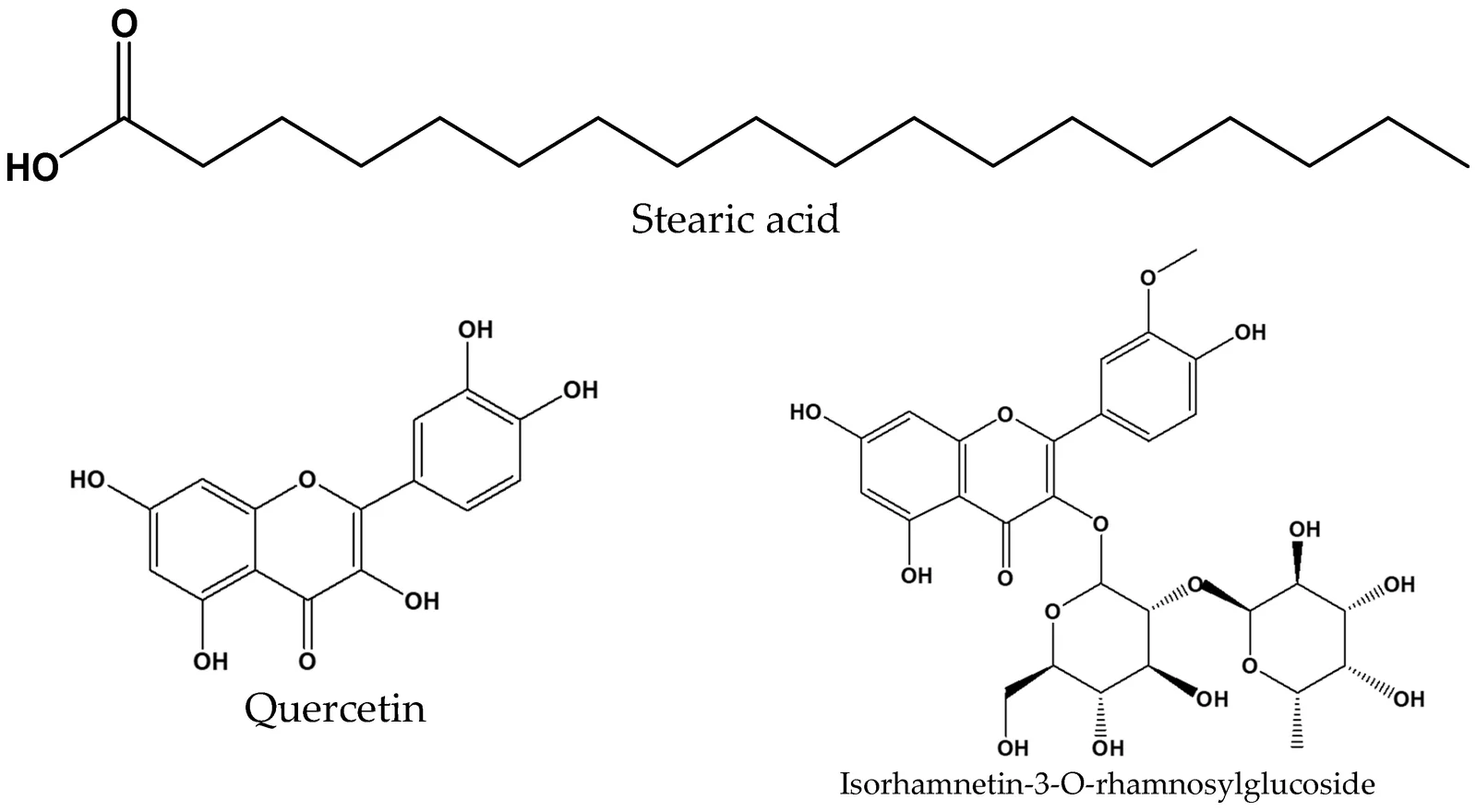

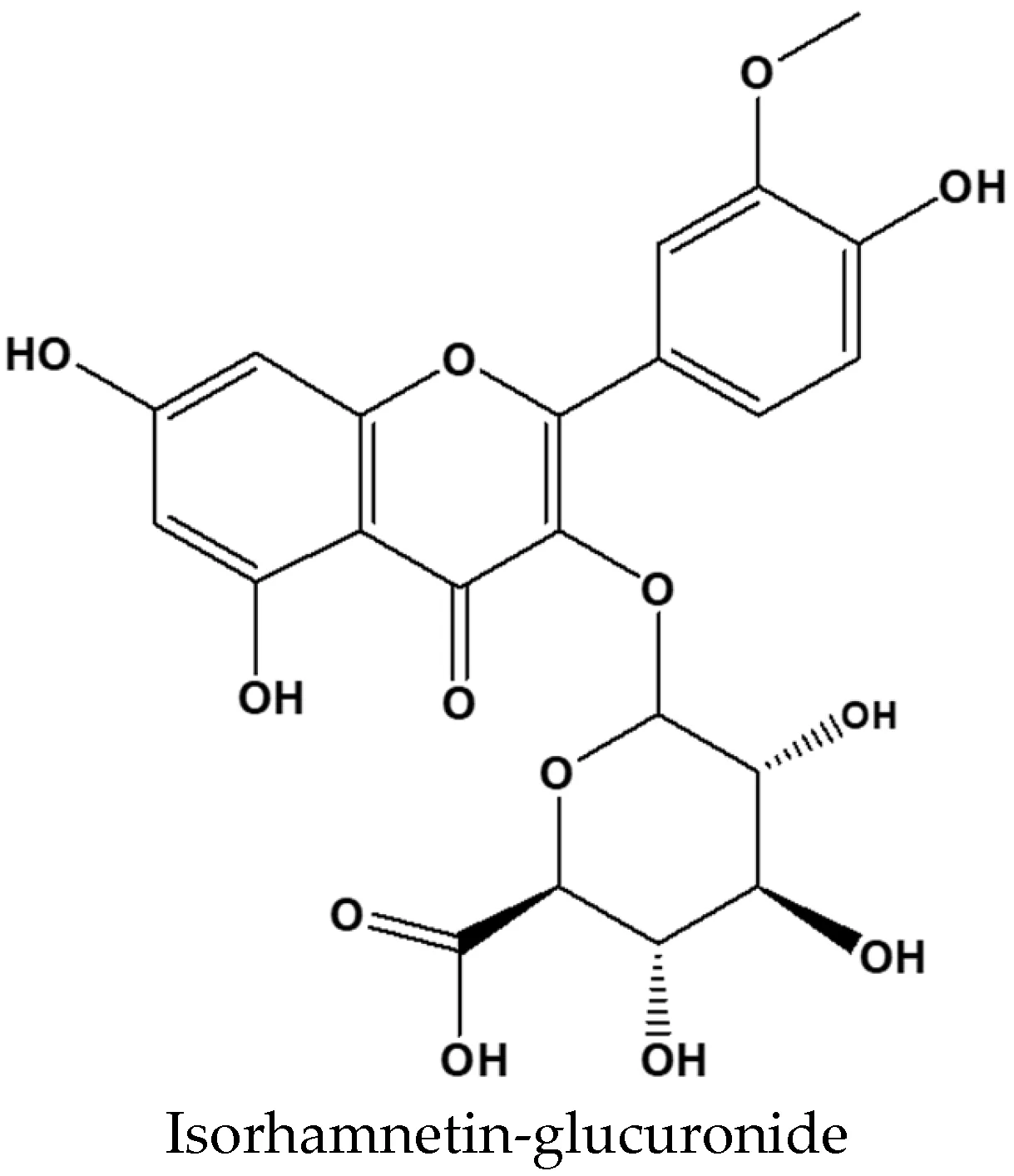
Structures of the four compounds identified from the Nigerian and South African *L. micranthus* DCM extracts using the Mass Bank spectral library and literature references.

For DCM UPLC-MS chromatogram, four distinct compounds (Table 1 and Figure 4) were identified from specific peaks, each characterised by unique retention time (Rt), molecular formulae, molecular masses, and mass fragmentation patterns. These compounds and their respective features are discussed below.

In the chromatographic analysis, Peak 3 in the chromatogram corresponded to stearic acid, characterised by an Rt of 9.11 min. Its molecular formula is C_18_H_36_O_2_, with a molecular mass of 285.2019. The mass fragmentation pattern showed peaks at 149, 241, 285, and 286. Unlike most saturated fatty acids that elevate low-density lipoprotein (LDL) cholesterol, stearic acid is a good cardiovascular and lipid regulator [21].

Moving to Peak 7, quercetin was identified with an Rt of 11.53 min. Its molecular formula is C_15_H_10_O_7_, and it has a molecular mass of 301.1397. Mass fragmentation displayed peaks at 93, 121, 149, 301, and 302. Quercetin falls into the category of flavonoids and is known for its versatile health benefits, including antioxidant and anti-inflammatory effects, along with potential anticancer properties [22].

Isorhamnetin-3-O-rhamnosylglucoside, identified as peak 8 with an Rt of 13.32 min, is characterised by the formula C_28_H_32_O_16_ and a molecular mass of 625.2670. Its mass fragmentation exhibited peaks at 243, 255, 271, 285, 299, and 302. As a flavonoid, isorhamnetin-3-O-rhamnosylglucoside is associated with potential antioxidant, anti-inflammatory, antimicrobial activities and cytoprotective activity [23]. Lastly, isorhamnetin-glucuronide, resolved at Peak 13 with an Rt of 15.83 min, has the molecular formula C_22_H_20_O_13_ and a molecular mass of 493.3512. Its mass fragmentation spectrum included peaks at 243, 255, 271, 299, and 302. It is an *O*-methylated flavonol glucuronide with strongest direct evidence for anti-inflammatory activity. In LPS-stimulated RAW264.7 macrophages, isorhamnetin-3-O-glucuronide increased heme oxygenase-1 (HO-1) expression and suppressed activation of the N-terminal kinase (JNK) and p38 mitogen-activated protein kinase (MAPK) pathways [24].

In perspective, from the 14 peaks that were detected in the UPLC-MS chromatogram analysis of the NGDCM and SADCM extracts from *L. micranthus*, four distinct compounds were revealed with unique retention times, molecular formulae, molecular masses, and mass fragmentation patterns. The four compounds should serve as a benchmark of the phytochemicals found in the dichloromethane extracts of Nigeria and South African *L. micranthus*, thus shedding lights on the pharmacological relevance of the plant.

**Table 1:** Identified compounds from NGDCM and SADCM *L. micranthus* from ultra-high-performance liquid chromatography quadrupole Time-of-Flight tandem mass spectrometry (UPLC-QTOF-MS).

Peak #	Rt (Mins)	Formula	Molecular Mass	Mass Fragmentation	Identified Compound	Source	Ref
3	9.11	C_18_H_36_O_2_	285.2019	149, 241, 285, 286.	Stearic acid	NG/SA	[25]
7	11.53	C_15_H_10_O_7_	301.1397	93, 121, 149, 301, 302.	Quercetin	NG/SA	[26]
8	13.32	C_28_H_32_O_16_	625.2670	243, 255, 271, 285, 299, 302	Isorhamnetin-3-O-rhamnosylglucoside	NG/SA	[27]
13	15.83	C_22_H_20_O_13_	493.3512	243, 255, 271, 299, 302	Isorhamnetin-glucuronide	NG/SA	[27]

### 2.6. Phytochemical Fingerprinting of NGMeOH vs. SAMeOH by UPLC-QTOF-MS

**Figure 5:**
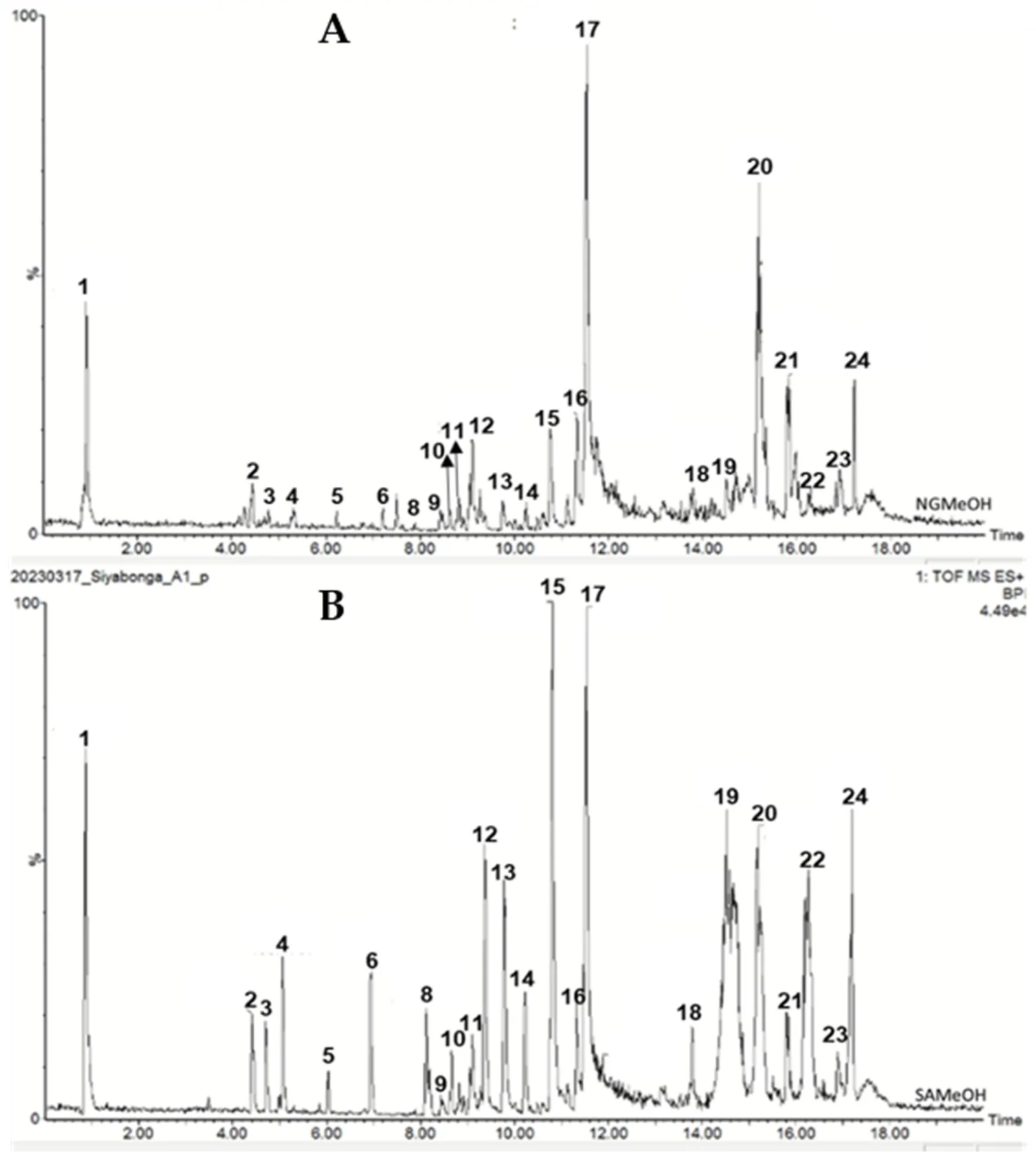
The total ion chromatograms of (**A**) NGMeOH and (**B**) SAMeOH under the positive ionization mode.

The UPLC-QTOF-MS analysis identified a total of 24 peaks in both NGMeOH and SAMeOH extracts (Figure 5). Despite sharing the same number of peaks, these extracts exhibit minor distinctive features. Notably, NGMeOH displayed a unique peak at 7.24 min, suggesting the presence of a compound absent in SAMeOH extract.

Furthermore, peaks at 8.41 min (Peak 9) demonstrated the lowest intensity in both extracts. Conversely, specific peaks at 4.41 min (Peak 2), 4.62 min (Peak 3), 5.06 min (Peak 4), 6.95 min (Peak 6), 8.12 min (Peak 8), 9.37 min (Peak 12), 9.79 min (Peak 13), 10.19 min (Peak 14), 10.81 min (Peak 15), 14.53 min (Peak 19), and 16.27 min (Peak 22) exhibited moderate intensity in SAMeOH but low intensity in NGMeOH. The peak with the highest intensity, observed at 11.53 min (Peak 17), consistently appeared in both extracts. This pointed to the presence of a shared major marker compound, mirroring the results obtained for both the Nigerian and South African DCM extracts.

Discrepancies in peak intensity and the presence or absence of peaks in crude extracts are not always apparent and necessitate the application of multivariate data analysis approaches for a thorough scrutiny. The absence of the peak at 7.24 min in SAMeOH implied a lower-to-zero concentration of the corresponding compound compared to NGMeOH. Conspicuously, the major compound at 11.53 min (Peak 17) exhibited a similar high abundance in both extracts, indicating an identical concentration of the corresponding compound in each. The identification of a unique compound in NGMeOH at 7.24 min raised questions about its potential pharmacological or toxicological impact in the quality control of mistletoe raw materials and commercial products, particularly when compared to its South African counterpart. Therefore, further investigation is crucial to identify, isolate, and elucidate the structure of this compound.

However, differences observed between the two extracts could be attributed to variations in geographic conditions [27]. For detailed peak patterns and intensities, refer to Figure 5 in the provided chromatograms. Over and above, the SAMeOH sample indicated moderately higher compound concentrations than the NGMeOH, except for the compound peak 15 that resolves at an Rt of 11.33 min.

### 2.7. Identification of Marker Compound Peaks in NGMeOH vs. SAMeOH

**Figure 6:**
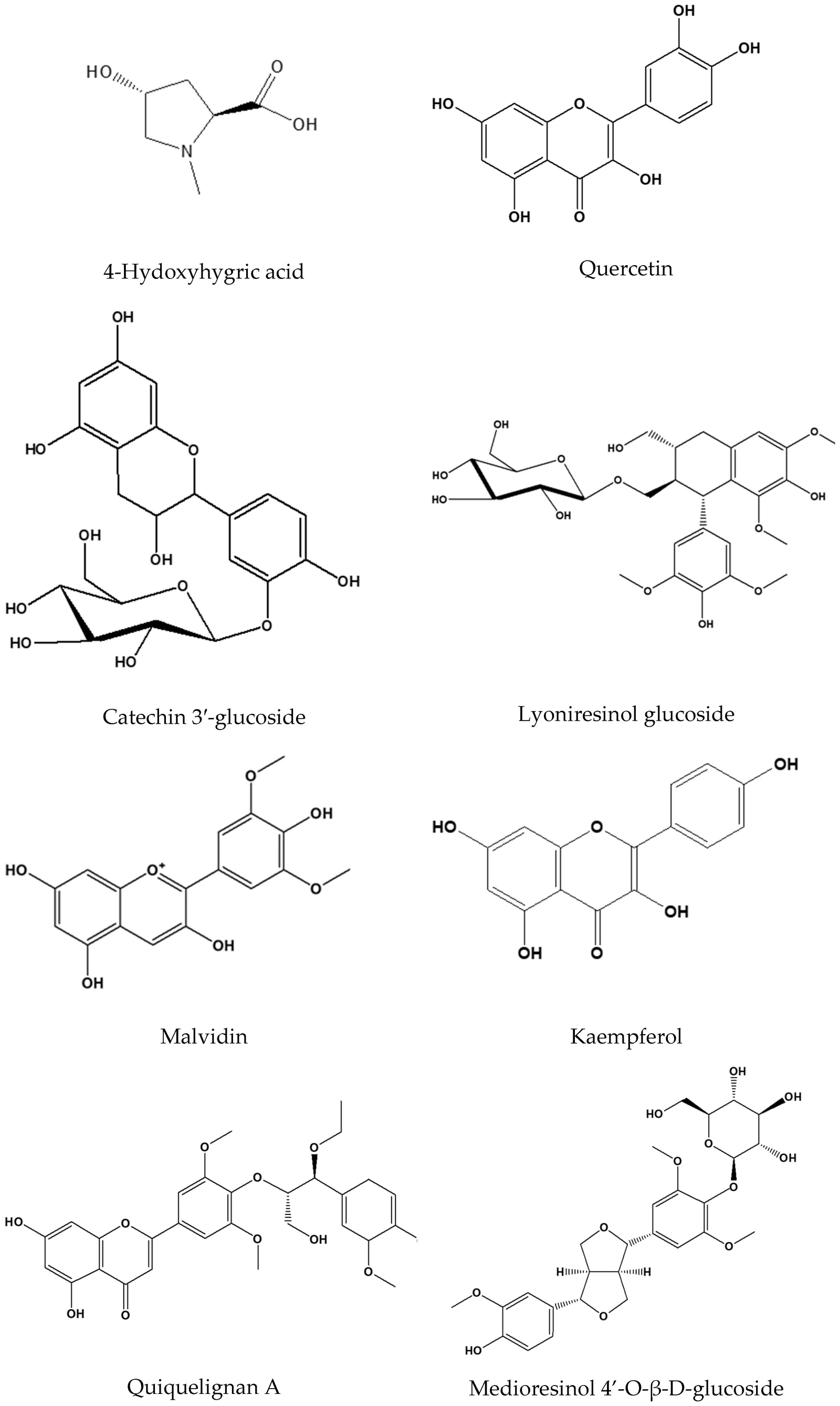

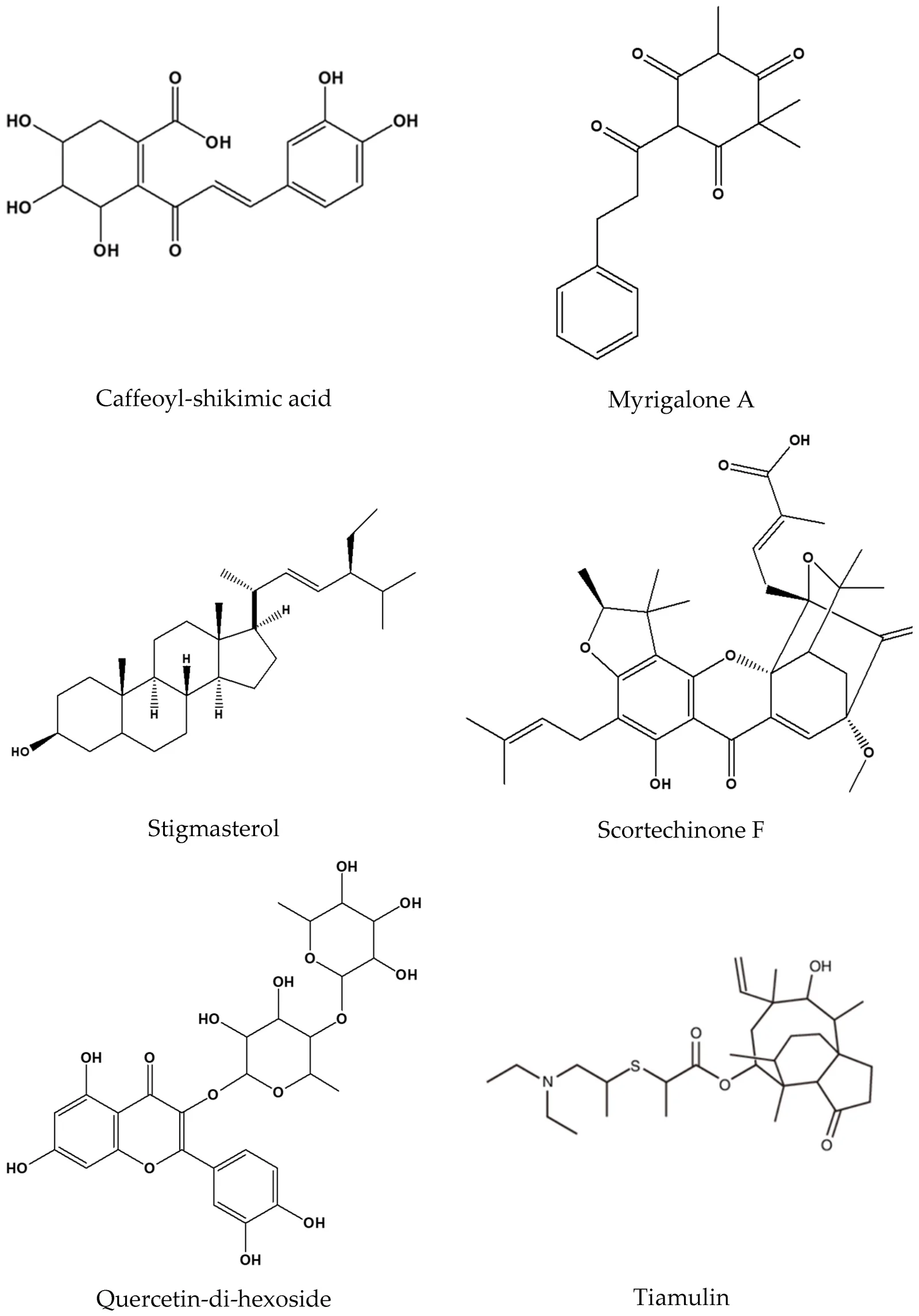
Structures of selected compounds proposed to be present in the NGMeOH and SAMeOH extract of *L. micranthus* as identified using spectral libraries and literature references.

In the analysis of NGMeOH and SAMeOH extracts using UPLC-QTOF-MS for compound identification (Table 2, Figure 6), 4-hydroxyhygric acid (peak 1) and quercetin (peak 3) were detected at Rt of 0.86 and 4.89 min, respectively based on accurate mass data. Their molecular formulae were confirmed as C_6_H_11_NO_3_ and C_15_H_10_O_7_ and they had fragmentation ions of 60, 102, 128, 146, and 153 and 165, 229, 275, 257, and 303.

Catechin-3′-glycoside (Peak 4) was detected at 5.06 min in the chromatogram. It has a molecular formula of C_21_H_22_O_11_ and a molecular mass of 453.1707. The mass fragmentation pattern featured peaks at 147, 257, 273, 291, and 453, aligning it with the class of non-protein imino acids. These compounds are recognised for their diverse biological activities, including insect-growth inhibition/antifeedant activity and a role in plant chemical defence [28].

Lyoniresinol-hexoside (Peak 8), at Rt of 8.53 min, was characterised with a molecular formula of C_28_H_38_O_13_ and a molecular mass of 593.2773. The mass fragmentation spectrum exhibited peaks at 205, 373, 389, 403, 421 and 583, indicating its classification as a lignan glycoside. Lignan glycosides are characterised with potential antioxidant and antiproliferative relevance [29].

With a retention time of 8.72 min, malvidin (Peak 9) was identified with a molecular formula of C_17_H_15_O_7_^+^ and a molecular mass of 333.1661. Its mass fragmentation pattern indicated 270, 287, 299, 315 and 333 peak ions. This compound falls into the category of flavonoids known for a plethora of biological activities, including anti-inflammatory and therapeutic effects [30,31].

With a retention time of 9.11 min, kaempferol (Peak 10) has a molecular mass of 285.2019 and mass fragmentation peaks at 153, 165, 177, 213 and 269. As a flavonoid with known antioxidants and anti-inflammatory properties, the role of kaempferol in human health management is vital [32].

Peak 13, resolved at 10.2 min, was characterised with a molecular formula of C_29_H_32_O_11_ and molecular weight of 557.1980; its fragmentation pattern afforded ions at 79, 149, 332, 548, 570 and 985. The metabolite was tentatively annotated as quiquelignan A, considered as a putative candidate based on accurate mass. However, quiquelignan A has previously been isolated from *Calamus quiquesetinervius* rather than mistletoe, and its occurrence in viscum requires confirmation using an authentic standard and/or orthogonal spectroscopic data [33]. The primary pharmacological study on quiquelignan A, indicated antioxidant, anti-inflammatory and antiplatelet-related activities [34]. The identification of peaks 14, 15, 16, 17, 19, 20 and 21 are summarised in Table 2.

In brief, the UPLC-QTOF-MS analysis of NGMeOH and SAMeOH extracts from *L. micranthus* revealed the presence of compounds belonging to diverse chemical classes and each compound holds promise for various biological activities for the management of human ailments using the plant extracts.

While these findings provide valuable insights into the chemical composition of *L. micranthus*, it is crucial to acknowledge the limitations in compound identification due to the scarcity of research on NG and SA *L. micranthus*, particularly concerning dichloromethane (DCM) extracts. Further studies are recommended to deepen our understanding of the pharmacological significance of these compounds and their potential applications in various fields.

Overall, this study contributes to the knowledge of the chemical constituents of *L. micranthus* and lays the foundation for future research to explore the full spectrum of their biological activities and potential therapeutic benefits.

**Table 2:** Identified compounds from NGMeOH and SAMeOH *L. micranthus* using ultra-high-performance liquid chromatography quadrupole Time-of-Flight tandem mass spectrometry (UPLC-QTOF-MS). Peaks were recorded in [M+H]^+^ mode.

Peak #	Rt (Mins)	Formula	Molecular Mass	Mass Fragmentation	Identified Compound	Source	Ref
1	0.86	C_6_H_11_NO_3_	146.0801	60, 102, 128, 146	4-Hydroxyhygric acid	NGMeOH, SAMeOH	[35]
3	4.89	C_15_H_10_O_7_	303.51	153, 165, 229, 275, 257, 303.	Quercetin	NGMeOH, SAMeOH	[36]
4	5.06	C_21_H_22_O_11_	453.1707	147, 257, 273, 291, 453	Catechin 3′-glucoside	NGMeOH, SAMeOH	[37]
8	8.53	C_28_H_38_O_13_	583.1804	205, 373, 389, 403, 421, 583	Lyoniresinol-hexoside	NGMeOH, SAMeOH	[38]
9	8.72	C_17_H_15_O_7_^+^	333.1661	270, 287, 299, 315, 333	Malvidin	NGMeOH, SAMeOH	[39]
10	9.11	C_15_H_10_O_6_	285.2019	153, 165, 177, 213, 259, 269	Kaempferol	NGMeOH, SAMeOH	[39]
13	10.25	C_29_H_32_O_11_	557.1980	79, 149, 332, 548, 570,	Quiquelignan A,	NGMeOH, SAMeOH	[40,41]
14	10.81	C_37_H_60_O_10_	661.2272	105, 149, 235, 661, 663	Medioresinol 4’-O-β-D-glucoside	NGMeOH, SAMeOH	[42]
15	11.33	C_16_H_16_O_8_	335.1226[M-H]-	149, 335, 357, 661, 850	Caffeoyl-shikimic acid	NGMeOH, SAMeOH	[43]
16	11.53	C_18_H_20_O_4_	301.1397	135, 153, 209, 255, 286	Myrigalone A	NGMeOH, SAMeOH	[44]
17	13.79	C_34_H_40_O_9_	593.2773	277, 289, 381, 437, 495, 564	Scortechinone F	NGMeOH, SAMeOH	[45]
19	15.19	C_29_H_48_O	413.2656	135, 147, 215,241, 255, 395, 413	stigmasterol	NGMeOH, SAMeOH	[46]
20	15.78	C_28_H_47_NO_4_S	494.3512	119,163,193, 476	Tiamulin	NGMeOH, SAMeOH	[47]
21	15.99	C_27_H_30_O_17_	628.3732	103, 121, 229, 217, 275, 303, 465, 627	Quercetin-di-hexoside	NGMeOH, SAMeOH	[48]

### 2.8. Antibacterial Activity of Plant Extracts Against P. aeruginosa, S. aureus, E. coli, and B. cereus Strains

The population of antibiotic-resistant harmful bacteria is rapidly increasing worldwide. Consequently, there is a critical need to explore new antimicrobial agents sourced from medicinal plants to counteract these resilient pathogens and prevent the emergence of untreatable bacterial infections, as emphasised by Bandow and co-workers in 2003 [18]. Hence, ongoing research into potential treatments for these bacteria is indispensable. Furthermore, the study results (Table 3), employing four distinct extracts, have yielded compelling evidence. The test results indicate consistent effects across all extracts when applied to diverse bacterial strains. It is notable that *Pseudomonas aeruginosa*, *Staphylococcus aureus*, *Bacillus strains*, *and Escherichia coli* all exhibited a uniform minimum inhibitory concentration (MIC) value of 1.5 × 10^−2^ mg/mL for each extract. Worth mentioning is the distinctive behaviour of *Streptococcus pyogenes*, which manifested a distinct MIC value of 7.5 × 10^−3^ mg/mL across all the extracts.

### 2.9. In-Extract Compounds Proposed to Be Responsible for the Antibacterial Potentials of the Investigated Extracts

The identification of compounds responsible for the antibacterial potential of plant extracts is a crucial aspect of understanding their therapeutic properties. Several studies have proposed various compounds in plant extracts that exhibit antibacterial activity.

Alkaloids are nitrogen-containing compounds found in many plants and are known for their diverse biological activities, including antibacterial properties. Studies have identified alkaloids in plant extracts that demonstrate antibacterial potential [49].

Flavonoids are a class of polyphenolic compounds present in various plant species. They have been recognised for their antioxidant and antibacterial activities. Research has suggested that flavonoids contribute to the antibacterial effects of certain plant extracts [50,51].

Terpenoids, including essential oils, are aromatic compounds found in many plant extracts. They have been investigated for their antibacterial properties. Some studies have highlighted the effectiveness of terpenoids against bacterial pathogens [52].

Phenolic compounds, such as tannins and phenolic acids, are widely distributed in plant extracts. These compounds have been associated with antibacterial activity, acting through various mechanisms [53].

Saponins are glycosides with soapy characteristics found in certain plant extracts. Research suggests that saponins possess antibacterial properties and can disrupt bacterial cell membranes [54].

These references provide insights into the diverse array of plant compounds that have been investigated for their antibacterial potentials. However, it is important to note that the specific antibacterial activity may vary depending on the plant species, extraction methods, and bacterial strains tested [55,56].

## 3. Discussion

The improved extract yield in the case of SADCM and SA MeOH, may be explained in terms of the cavitation effects caused by high-intensity ultrasound [57]. Ultrasound-assisted solvent extraction is a technology that uses high-intensity, high-frequency sound waves and solvents to extract compounds from various matrices. The physical and chemical properties of the materials subjected to ultrasound are altered because of the propagation and interaction of sound waves as they disrupt plant cell walls, facilitating the release of extractable compounds and enhancing mass transport of solvent from the continuous phase into plant cells. The best extraction procedure and solvent must be chosen based on sample matrix parameters, analyte chemical properties, matrix–analyte interaction, efficiency, and desired characteristics [58,59]. The extract yield of MeOH extracts was dominant compared to the extract yield of DCM extracts, possibly due to the higher polarity of MeOH and elevated extraction temperature.

The comprehensive analysis of mistletoe tree extracts from Nigeria and South Africa involves a multifaceted exploration of their phytochemical composition and antibacterial potential, employing sophisticated chromatographic techniques such as TLC and UPLC-QTOF-MS. The TLC analysis of *L. micranthus* extracts revealed distinct colours under different UV wavelengths, indicating the presence of antioxidants like anthracene derivatives, coumarins, and phenolic carboxylic acids. Further investigations with PEG and MeOH:H_2_SO_4_ (9:1 *v*/*v*) derivatisation highlighted specific compounds, emphasising the need for continued exploration into mistletoe extracts’ unique properties under different conditions.

Transitioning to UPLC-MS analysis, the spectra unveiled common peaks in both NGDCM and SADCM extracts, with a unique peak at 13.32 min in SADCM, that suggested a compound absent in NGDCM. Major compounds at 11.53 and 15.19 min were consistent in both extracts, indicating shared components with potential pharmacological significance. Analysis of methanol extracts (NGMeOH vs. SAMeOH) identified 14 peaks in each, with NGMeOH exhibiting a unique peak at 7.24 min. Despite the challenges, the detailed information in Table 2 and Table 3, along with structural insights in Figure 4 and Figure 6, formed a foundation for future research and addressed the scarcity in the scientific literature for African *L. micranthus*.

The antibacterial activity assessment (Table 3) against prevalent strains demonstrated a consistent but potent minimum inhibitory concentration (MIC) of 1.5 × 10^−2^ mg/mL across mistletoe extracts compared to ciprofloxacin (MIC = 3.9 × 10^−3^), suggesting potent antibacterial effects. Notably, *Streptococcus pyogenes* exhibited a distinctive MIC of 7.5 × 10^−3^ mg/mL. The MIC values of the current study corroborated those reported by previous study. Accordingly, MIC of 0.37–0.75 were reported for extracts *V. continuum* [60], 1.25 mg/mL for extracts of *V. cruciatum* [61] and 1.25–5 mg/mL for related *Loranthaceae* extracts [62].

This aligns with the urgent need to explore alternative antimicrobial sources amid rising antibiotic resistance. This study unravelled compounds like alkaloids, flavonoids, terpenoids, phenolic compounds, and saponins, associated with antibacterial potential, highlighting their variability based on plant species, extraction methods, and bacterial strains. This holistic approach provided insights into mistletoe extracts’ potential therapeutic applications against resistant bacteria, promising further exploration and development of alternative antimicrobial agents.

## 4. Materials and Methods

### 4.1. Plant Collection, Comminution, and Extraction

A mistletoe plant was identified by a botanist at Obafemi Awolowo University in Nigeria (latitude 7°31′14.7612″ N, longitude 4°31′49.1340″ E). The plant, weighing 2.0 kg in powder form, underwent processing at the Collect and Curate Initiative Centre of the Pharmacognosy Department. On 1 March 2019, it was purchased with the sample voucher specimen NGMTT01. Leaf samples from South Africa were collected in the Orchards residential area, Pretoria (25°38′57.9″ S, 28°05′32.6″ E), during autumn. These were identified by a traditional healer and SANBI and labelled with voucher specimen SAMTT01. Both vouchers were deposited in the Herbarium of the Pharmaceutical Sciences Department at Sefako Makgatho Health Sciences University.

The plant sample underwent drying, grinding using a Polymix Laboratory Dry Mill Drive unit, and was stored in brown paper bags till further use. For extraction, 100 g of powdered plant material was placed in 100 mL of dichloromethane (DCM) in a 250 mL Erlenmeyer flask and subjected to ultrasonic extraction at the boiling point of DCM for 30 min. After filtration with Whatman number 4 filter paper, the process was repeated twice, and the combined filtrate was rotary evaporated to yield the dry mass of the DCM extract. Another 100 g of plant powder was separately extracted with 100 mL of methanol using the same extraction procedure, and the percentage yield was calculated using equation 1 below.

% Yield of dry extract = W1/W2 × 10(1)

where W1 is dry extract weight after extraction and W2 is the weight of the dry starting material weight.

Following extraction, 1.0 mg of each extract was dissolved in the suitable solvents. The solutions were filtered, spotted on a TLC plate, and dried. The methanol extract plate was developed using ethyl acetate: hexane (1:3 *v*/*v*), and the DCM extract plate used hexane: acetone: ethyl acetate (8:1:1 *v*/*v*/*v*). Chromatograms were initially viewed without chemical treatment under a short wavelength of 254 nm and long wavelength of 366 nm. For the spraying procedure, each developed TLC plate was placed on filter paper, and a sprayer filled with a 0.2 mg/mL DPPH solution was used from around 15 cm using a rubber ball/pump.

### 4.2. Thin-Layer Chromatography Analysis

Following the extraction procedure, 1.0 g/5.0 mL was spotted on the TLC plate. Chromatography was conducted in the following solvent systems: EtOAc: toluene (1:2 *v*/*v*) for the methanol extract and dichloromethane extracts and hexane: acetone: ethyl acetate (8:4:4 *v*/*v*/*v*). After developing the TLC plate in their respective mobile phases, the chromatograms were viewed initially without chemical treatment, under UV 254 and UV 366 nm light. For the spraying procedure, the developed, dried TLC plate or sheet was positioned on a sheet of filter paper beneath a fume. The sprayer was filled with 5 to 10 mL of derivatising solution and sprayed from around 15 cm using a rubber ball/pump.

### 4.3. Ultra-High-Performance Liquid Chromatography Quadrupole Time-of-Flight Tandem Mass Spectrometry (UPLC-QTOF-MS) of the Plant Extracts

Briefly, 1.0 mg/mL of each extract was separately introduced by full-loop injection (1.0 μL) into a UPLC (Waters Acquity chromatographic system, Waters, Milford, MA, USA). The MS, UPLC and gradient system used are displayed in Table 4.

### 4.4. Antibacterial Activity of Plant Extracts Against P. aeruginosa, S. aureus, E. coli, and B. cereus Strains

The selected bacterial strains, namely *P. aeruginosa*, *S. aureus*, *E. coli*, and *B. cereus*, were acquired from the Next Gen Health unit at the Council for Scientific and Industrial Research (CSIR) in South Africa. These bacterial strains were maintained as glycerol stocks at −80 °C until needed for use. The strains were prepared in Mueller–Hinton (MH) medium during the minimum inhibitory concentration (MIC) assay and incubated at 37 °C to obtain active bacterial cultures. One or two colonies were often transferred to sterile distilled water to achieve an absorbance (OD600 nm) of 0.1. The cell suspension was adjusted to reach a 0.5 McFarland standard equivalent.

The MIC of the plant-derived chemical compounds was determined using a method outlined by [63,64] with some minor adjustments. Initially, stock solutions of the compounds at a concentration of 1 mg/mL were prepared. Subsequently, 100 microliters (μL) of a bacterial growth medium (MH broth) were added to each well of a 96-well microtiter plate. Following this, 100 μL of each chemical compound was added to the first row of wells in duplicate. Serial dilutions were performed from row A to row H, resulting in decreasing concentrations ranging from 0.25 mg/mL to 0.001 mg/mL.

Next, 100 μL of standardised bacterial strains (measured at OD 600 nm = 0.08–0.1) were added to each well. Each plate was set up with both positive and negative control wells. Ciprofloxacin, a known antibiotic, served as the positive control at concentrations of 1 mg/mL and 0.01 mg/mL, while 100 μL of a solution containing 1% dimethyl sulfoxide (DMSO) was employed as the negative control. The plates were then incubated at 37 °C for 24 h. Following the incubation period, 40 μL of a solution containing *p*-iodonitrotetrazolium violet (INT) at a concentration of 0.20 mg/mL was added to each well and incubated again at 37 °C for 30 min. The MIC value for each chemical compound was determined visually and recorded. MIC was defined as the lowest concentration of the compounds where no visible growth of the test bacteria was observed. The antibacterial experiment was conducted in three separate repetitions.

## 5. Conclusions

The in-depth analysis of mistletoe tree extracts from Nigeria and South Africa through advanced chromatographic techniques sheds light on their phytochemical composition and antibacterial potential. The diverse insights obtained from TLC and UPLC-QTOF-MS highlighted the unique fingerprints of the extracts and underscored the need for continued exploration into their distinct properties under varying conditions. The identification of common and unique compounds, especially the noteworthy 13.32 min peak in SADCM and the 7.24 min peak in NGMeOH, sets the stage for future research to unveil their potential pharmacological significance. The consistent MIC values against prevalent bacterial strains, except for Streptococcus pyogenes, emphasised the promising antibacterial effects of these extracts, aligning the imperative need to seek alternative antimicrobial sources in the face of increasing antibiotic resistance. The study’s comprehensive approach, linking phytochemical profiles with antibacterial activity, has contributed valuable insights into the potential therapeutic applications of mistletoe tree extracts, urging further exploration and development of alternative antimicrobial agents.

## Figures and Tables

**Table 3:** Activity of four mistletoe tree extracts against isolates of bacteria strains using the MIC method.

	Minimum Inhibitory Concentration (MIC * in mg/mL)				
	*P. aeruginosa*	*S. pyogenes*	*S. aureus*	*E. coli*	*B. cereus*
SAMeOH	1.5 × 10^−2^	7.5 × 10^−3^	1.5 × 10^−2^	1.5 × 10^−2^	1.5 × 10^−2^
NGMeOH	1.5 × 10^−2^	7.5 × 10^−3^	1.5 × 10^−2^	1.5 × 10^−2^	1.5 × 10^−2^
SADCM	1.5 × 10^−2^	7.5 × 10^−3^	1.5 × 10^−2^	1.5 × 10^−2^	1.5 × 10^−2^
NGDCM	1.5 × 10^−2^	7.5 × 10^−3^	1.5 × 10^−2^	1.5 × 10^−2^	1.5 × 10^−2^
*Ciprofloxacin*	3.9 × 10^−3^	3.9 × 10^−3^	3.9 × 10^−3^	1.56 × 10^−2^	3.9 × 10^−3^

* Each value is expressed as mean of (n = 3) triplicate experiments.

**Table 4:** The UPLC-QTOF-MS conditions used to analyse the plant extracts.

MS CONDITIONS		
Detector	Waters^®^ Synapt G2QTOF	
Calibration mass range	50–1200 m/z	
Capillary voltage	ESI+ 2.6 KV; ESI− 2.4 KV	
Ionization mode	Both ESI+ and ESI−	
Source temperature	120 °C	
Sampling cone	20 V	
Extraction cone	4.0 V	
Desolvation temperature	300 °C	
Cone gas flow	20.0 L/Hr	
Desolvation gas flow	600.0 L/Hr	
Data management	MassLynx^TM^ (version 4.1 UNIFI)	
UPLC CONDITIONS		
System	Waters Acquity uplc	
Column	Kinetex^®^ 1.7 µm EVO C18 100 Å (2.1 mm ID × 100 mm length)	
Injection volume	5 µL	
Column temperature	50 °C	
Sample temperature	8 °C	
Flow rate	0.3 mL/min	
Mobile phase A	Water + 0.1% formic acid	
Mobile phase B	Acetonitrile 0.1% formic acid	
GRADIENT		
Time (min)	%A	%B
Initial	97.0	3.0
0.10	97.0	3.0
14.00	0	100.00
16.00	0	100.00
16.50	97.0	3.0
20.00	97.0	3.0

## Data Availability

The data generated in this study are part of the manuscript.

## Abbreviations

The following abbreviations were used in this manuscript:

UPLC-MS	Ultra-High-Performance Liquid Chromatography–Mass Spectrometry
TLC	Thin-Layer Chromatography
DCM	Dichloromethane
MeOH	Methanol
MIC	Minimum Inhibitory Concentration
NGG	Nigerian Green
NGB	Nigerian Brown
UV	Ultraviolet
PEG	Polyethylene Glycol
NP	Natural Product
SADCM	South African Dichloromethane
NGDCM	Nigerian Dichloromethane
EtOAc	Ethyl acetate
NGMeOH	Nigerian Methanol
SAMeOH	South African Methanol
QTOF	Quadrupole Time-of-Flight
NGMTT	Nigerian Mistletoe tree
SANBI	South African National Biodiversity
SAMTT01	South African Mistletoe tree
DPPH	Diphenyl-dipicryl hydrazyl
CSIR	Council for Scientific and Industrial Research
MH	Mueller–Hinton
INT	*p*-Iodonitrotetrazolium violet
